# Supplementary material for: Infection patterns of endemic human coronaviruses in rural households in coastal Kenya
Source: Wellcome Open Res. 2021 Feb 9;6:27. [Version 1] doi: 10.12688/wellcomeopenres.16508.1 (PMC8669777; doi:10.12688/wellcomeopenres.16508.1)
Supplement: Peer review history

# Peer review: Reviewer response for version 1

**DOI:** 10.21956/wellcomeopenres.18181.r46783

**Published:** 2021-12-13

**Authors:** Pragya D. Yadav

**Affiliations:** 1 Indian Council of Medical Research, National Institute of Virology, Pune, Maharashtra, India

I have read the manuscript titled “Infection patterns of endemic human coronaviruses in rural households in coastal Kenya” by Nyaguthii *et al*. However, there are many flaws associated with the study design, methodology, analysis and interpretations as below.    

1. This is more of retrospective study. The results on the prevalence of Human coronaviruses have been already published by the authors (Reference no. 8).
2. It is not clear from the reference no. 8 and 10, how the authors have come to the conclusion of screening 483 individuals from 47 households. What was the exclusion, inclusion criteria adapted to select the study population?
3. Interval of specimen collection from study subjects differs in methodology of abstract and main text.
4. Total number of samples screened (n=16,918) for 483 subjects as per the study protocol (once a week for four weeks and twice a week for next five months) is less. Please provide the justification for the same.
5. Why screening of HCoV-HKU1 was not included in the study? Sipulwa *et al.*, 2016 have demonstrated the prevalence of HCoV-HKU1 in Kenya during 2009-2012. ^1^
6. What was the viral RNA copy number in high peak viral load and low peak viral load in virus shedding pattern?
7. Apparently, the details related to re-infection is missing i.e., the time interval between the two infections, whether the subjects were tested negative before re-infection, the detailed laboratory diagnosis of re-infection cases.
8. Transmission of HCoV in households is not clear.
9. There is restricted access to the supplementary data; hence it could not be analyzed.
10. Please cite the study of Sipulwa et al., 2016 on the prevalence of Human coronaviruses in Kenya during 2009-2012.
11. Please remove conclusive remark on SARS-CoV-2 from abstract.

Is the work clearly and accurately presented and does it cite the current literature?

Partly

If applicable, is the statistical analysis and its interpretation appropriate?

Yes

Are all the source data underlying the results available to ensure full reproducibility?

Partly

Is the study design appropriate and is the work technically sound?

Partly

Are the conclusions drawn adequately supported by the results?

Yes

Are sufficient details of methods and analysis provided to allow replication by others?

Partly

Reviewer Expertise:

Virology

I confirm that I have read this submission and believe that I have an appropriate level of expertise to confirm that it is of an acceptable scientific standard, however I have significant reservations, as outlined above.

## References

[1] : Molecular characterization of human coronaviruses and their circulation dynamics in Kenya, 2009-2012. *Virol J* .2016;13: 10.1186/s12985-016-0474-x 18 10.1186/s12985-016-0474-x 26833249PMC4736488

# Peer review: Reviewer response for version 1

**DOI:** 10.21956/wellcomeopenres.18181.r46787

**Published:** 2021-11-24

**Authors:** Dongsheng Hu

**Affiliations:** 1 School of Public Health, Shenzhen University Health Science Center, Shenzhen, China

1. ​​​​​​Please describe clearly how nasopharyngeal swabs were collected. The author's description in the abstract was inconsistent with that in the methods section. In addition, no matter which method was used to collect samples, the total number of samples should be greater than 16,918. Why were only 16,918 samples in this study?
2. For continuous variables, when were the mean (±standard deviation) and median (interquartile range (IQR)) used respectively?
3. The authors didn’t provide the hypothesis testing level of this study and it is suggested to add.
4. On the left side of page 5, lines 4 through 6, the author stated that the positive and negative samples were 1,274 and 15,645, respectively. According to this situation, the total number of samples should be 16,919; however, the total number of samples was 16,918 in the previous description. Please check it carefully.
5. In this study, the risk of secondary transmission was higher among index cases with high viral loads; however, it was not consistent with other studies. It is recommended to analyze the possible causes.

Is the work clearly and accurately presented and does it cite the current literature?

Yes

If applicable, is the statistical analysis and its interpretation appropriate?

Partly

Are all the source data underlying the results available to ensure full reproducibility?

Partly

Is the study design appropriate and is the work technically sound?

Yes

Are the conclusions drawn adequately supported by the results?

Yes

Are sufficient details of methods and analysis provided to allow replication by others?

Partly

Reviewer Expertise:

Epidemiology

I confirm that I have read this submission and believe that I have an appropriate level of expertise to confirm that it is of an acceptable scientific standard, however I have significant reservations, as outlined above.

# Peer review: Reviewer response for version 1

**DOI:** 10.21956/wellcomeopenres.18181.r42819

**Published:** 2021-03-12

**Authors:** Eleanor Gaunt

**Affiliations:** 1 Department of Infection and Immunity, The Roslin Institute, The University of Edinburgh, Edinburgh, UK

**Synopsis:**

 Nyaguthii and colleagues describe the screening of 483 individuals from 47 households for respiratory viruses over a 6 month period in the 2009-2010 respiratory syncytial virus (RSV) season. In this report, the detection data for human coronaviruses OC43, NL63 and 229E are described. There are some intriguing numbers on reinfection and duration of ‘shedding’ (as proxied using PCR positivity). The data are of interest because we have seen these types of studies in the developed world, but in this study, a household has, on average, over 10 individuals, and so these data provide a far more robust investigation of within household transmission. The data are thoroughly analysed, carefully interpreted, and the discussion is sensible and does not over-interpret.

**Major Comment:**

 There raw data linked in the manuscript is restricted access for ethical reasons, so I cannot review it. Is it not possible to anonymise the data and share it that way? (I appreciate that this may not be possible given how detailed the data are).

**Minor Comments:**

1. Methods: Although the study design is previously published, a sentence on how the clinical data were collected would be appropriate.
2. Methods: Why is HCoV-HKU1 not screened for?
3. Methods: RT-PCR should read RT-qPCR.
4. Methods: hMPV should be reannotated HMPV in line with ICTV guidance. Similarly throughout the manuscript hCoV and HCoV are used interchangeably; this should be HCoV throughout.
5. Methods: The paragraphs below the ‘Statistical analysis’ section are lacking headings.
6. Methods: Using PCR based detection as a proxy for shedding raises an eyebrow. As the approach can be expected to be consistent across the viruses, the relative measures of shedding are likely reasonable, but the raw numbers may not be. This is briefly commented on in the discussion but I feel that this could be elaborated.
7. Results: in baseline characteristics, for gender references ‘male’ is referred to when these were a minority in the study set. The majority population (female) should instead be referred to unless gender was unavailable/ not disclosed for some subjects.
8. Results: The sentence ‘Symptomatic individuals contributed to 240…of samples that tested negative’ is confusing to read and I am not really sure what this is saying – please rephrase for clarity.
9. Results: There are several places where ‘any HCoV type’ is referred to, but I am not sure whether this is referring to specifically mixed infections- in some cases, it seems to be. Can this be defined somewhere?
10. Figure 1: I cannot distinguish between open and closed dots, are they really different? (I am looking at a print out)
11. Results: For the duration of shedding, if I am interpreting Fig. 1 correctly, you have some examples where shedding occurs for over 3 months. That is worth a sentence or two! (Also in the discussion)
12. Results: Reinfection data describe occasions where individuals tested positive in sequential tests, but these were categorised as reinfection. On what basis was that judgement made? It seems counterintuitive.
13. Results: Reinfection data describe the frequencies of reinfection with each of the three HCoVs studied – do these frequencies differ from the overall frequencies?
14. Discussion: The abstract mentions SARS-CoV-2, but no parallels are made in the discussion – either take this out of the abstract or make an interpretation in the discussion.

Is the work clearly and accurately presented and does it cite the current literature?

Yes

If applicable, is the statistical analysis and its interpretation appropriate?

Yes

Are all the source data underlying the results available to ensure full reproducibility?

Partly

Is the study design appropriate and is the work technically sound?

Yes

Are the conclusions drawn adequately supported by the results?

Yes

Are sufficient details of methods and analysis provided to allow replication by others?

Partly

Reviewer Expertise:

RNA virology

I confirm that I have read this submission and believe that I have an appropriate level of expertise to confirm that it is of an acceptable scientific standard.
